# Hypothalamic POMC expression is required for peripheral insulin action on hepatic gluconeogenesis through regulating STAT3 in sepsis rats

**DOI:** 10.1111/jcmm.13449

**Published:** 2017-12-28

**Authors:** Bin Feng, Nannan Zhang, Kaipeng Duan, Bimin Shi

**Affiliations:** ^1^ Department of Endocrinology and Metabolism The First Affiliated Hospital of Soochow University Suzhou Jiangsu China; ^2^ Department of Cardiology The First Affiliated Hospital of Soochow University Suzhou Jiangsu China; ^3^ Department of General Surgery The First Affiliated Hospital of Soochow University Suzhou Jiangsu China

**Keywords:** hypothalamus, POMC, sepsis, peripheral insulin, hepatic gluconeogenesis

## Abstract

Liver injury and dysregulated glucose homoeostasis are common manifestations during sepsis. Although plenty of studies reported insulin could protect against multiple organ injuries caused by critical infections among patients, little was known about the precise mechanism. We investigated whether liver inflammatory pathway and central neuropeptides were involved in the process. In sepsis rats, hepatic IKK/NF‐κB pathway and STAT3 were strongly activated, along with reduced body weight, blood glucose and suppressed hepatic gluconeogenesis (GNG). Peripheral insulin administration efficiently attenuated liver dysfunction and glucose metabolic disorders by suppressing hypothalamic anorexigenic neuropeptide proopiomelanocortin (POMC) expression, hepatic NF‐κB pathway and STAT3 phosphorylation. Furthermore, knockdown of hypothalamic POMC significantly diminished protective effect of insulin on hepatic GNG and insulin‐induced STAT3 inactivation, but not inflammation or IKK/NF‐κB pathway. These results suggest that hepatic IKK/NF‐κB pathway mediates the anti‐inflammatory effect of insulin in septic rats, and peripheral insulin treatment may improve hepatic GNG by inhibiting STAT3 phosphorylation dependent on hypothalamic POMC expression.

## Introduction

Sepsis commonly happens in critical illness and usually causes severe complications, such as metabolic disorders, in which reduced feeding, increased energy expenditure and dysregulated glucose homoeostasis are recognized as hallmarks 1. Additionally, because of multiple functions of the liver, high levels of pro‐inflammatory cytokines and oxidative insults‐induced liver injury have critical effects on the outcome of sepsis patients 2. Depend on the severity and stage of sepsis, changes in glucose metabolism are complex 3. Notably, albeit sepsis is usually associated with hyperglycaemia, hypoglycaemia is observed in models of sepsis and thought to be associated with mortality of septic patients 4, 5, 6. Earlier studies reported that insulin treatment protects against multiple organ damages caused by sepsis and reduces mortality among critical patients 7. Although animal experiments have demonstrated NF‐κB pathway and glycogen synthase kinase (GSK)‐3β inhibition were involved 8, 9, 10, the precise molecular mechanisms underlying beneficial effects of insulin remain undefined.

Signal transducer and activator of transcription 3 (STAT3) has been demonstrated a putative role in regulating expression of hepatic gluconeogenic genes 11. Phosphorylated STAT3 is recruited to the promoter regions of G6Pase and PEPCK, leading to reduced gene expression. This is supported by the fact that liver‐specific STAT3 knockout resulted in increased expression of these genes, on the contrary, overexpression of active STAT3 in the liver caused reduced gene expression of PEPCK and G6Pase, as well as blood glucose 12, 13, 14. In addition, intraperitoneal administration of lipopolysaccharide (LPS) increased the phosphorylation of STAT3 in the liver 15.

Hypothalamus plays critical roles in the regulation of energy metabolism balance as well as the maintenance of glucose homoeostasis 16. Pro‐opiomelanocortin (POMC) is principally expressed in the arcuate nucleus (ARC) of the hypothalamus and the nucleus of the tractus solitarius (NTS) of the brainstem 17. The peptide precursor POMC can be further cleaved into α‐melanocyte‐stimulating hormone (α ‐MSH), an agonist for central melanocortin receptors expressed on secondary neuron populations, including melanocortin‐3 receptor (MC3R) and MC4R, to increase energy expenditure and suppress food intake. More importantly, insulin could regulate glucose metabolism through both the direct action on the liver and indirect modulation on the hypothalamus 14. However, precise molecular mediators remain obscure. Here, we explored the mechanisms by which long‐acting insulin analogue insulin glargine administration, in the context of LPS induced sepsis, contributes to liver injury and glucose metabolism disturbances. To this aim, we employed rat models of sepsis with hypothalamus‐specific POMC knockdown and further tested whether the detrimental impacts of sepsis on liver were diminished.

## Materials and methods

### Animals and treatments

Male Sprague‐Dawley rats (Experimental Animal Center of Nanjing Medical University, Jiangsu, China) ages 6 weeks were used in this study. Animals were housed in a 12‐hr light/dark cycle with free access to food and water throughout the study. Experimental protocols were by the Animal Care and Use Committee of Nanjing University (Approval ID: 2016000538) and conformed to the Guide for the Care and Use of Laboratory Animals of the U.S. National Institutes of Health. Rats were randomly assigned to different groups: Con, no treatment; LPS, intraperitoneally (i.p.) administration of LPS (10 mg/kg, E. coli, 055:B5, Sigma‐Aldrich, Shanghai, China); LPS+Insulin, 1 hr after LPS administration, rats received a subcutaneous (s.c.) injection of Insulin glargine (65 units/kg; Lantus, Sanofi‐Aventis, Paris, France); LPS+V, 1 hr after LPS administration, rats received a s.c. injection of vehicle (saline, 150 μl). After 24 hrs, the hypothalamus and liver were rapidly dissected and snap‐frozen in liquid nitrogen and stored at −80°C until use. In POMC knockdown experiments, 2 weeks before LPS injection, rats were bilateral injected with lentiviral POMC shRNA (LPS/KD) or matched control (LPS/V) to the ARC, respectively. One hour after LPS administration, rats of LPS/KD and LPS/V received a s.c. injection of vehicle or Insulin glargine (LPS/KD+Ins and LPS/V+Ins). At the end of the study, rats were subjected to intraperitoneal glucose, pyruvate and insulin tolerance tests (IPGTT, IPTT and ITT).

### Intra‐hypothalamic injections of lentiviruses

To knockdown POMC expression in ARC cells, the intra‐hypothalamic injection of lentiviral vector of shRNA against rat POMC or matched control (GenPharma Co., Ltd Shanghai, China) was performed. After confirming the interfering effect *in vitro*, the sequence of shRNA was determined as CUCUUCAAGAACGCCAUCA (5′ ‐3′). HEK 293T cells were cotransfected with target sequences to produce recombinant lentiviruses. 1 × 10^9^ particles/site of purified lentiviruses was used for each injection. The bilateral injection to the ARC was directed to the orientation of 3.3 mm posterior to the bregma, 9.0 mm below the surface of the skull and 0.3 mm lateral to midline by an ultra‐precise stereotax (Kopf Instruments, Tujunga, CA, USA). Purified lentivirus were injected over 10 min. using a 5‐μl Hamilton syringe connected to a microinfusion pump (World recision Instruments, Sarasota, FL, USA).

### IPGTT, IPTT and ITT

IPGTT (D‐glucose; 2.0 g/kg body weight) and IPTT (sodium pyruvate; 1.0 g/kg body weight) were performed on overnight fasted rats. Insulin sensitivity tests (Novolin R; 0.65 U/kg body weight) were performed on 5 hrs food deprived rats. Blood samples were collected *via* the tail vein. All compounds were intraperitoneally injected (i.p.) and blood glucose determined at the indicated time‐points.

### Biochemical measurements

Plasma samples were obtained from vena orbitalis posterior of rats. All biochemical determinations were performed using commercially available kits according to the manufacturer's instructions. Plasma insulin and IL‐6 were measured by ELISA (R&D Systems, Minneapolis, MN, USA); liver triglyceride levels were measured by the Triglyceride (TG) Quantification Kit (ab‐65336, Abcam, Cambridge, UK). Liver glycogen measurements were performed as a previously described procedure 18. Hepatic aspartate aminotransferase (AST) and alanine aminotransferase (ALT) activity were determined using specific kits according to the manufacturer's instructions (Jiancheng Bioengineering Institute, Nanjing, China). Plasma corticosterone levels were measured by RIA (MP Biomedicals, Irvine, CA, USA) according to the manufacturer's instructions. For α‐MSH content measurement, lysates of hypothalami were centrifuged and supernatants used for the quantification by ELISA (Phoenix Pharmaceuticals, Belmont, CA, USA).

### Hepatic ROS determination

*In situ* O_2_^−^ levels were measured with the oxidative fluorescent dye dihydroethidium (DHE; Beyotime Institute of Biotechnology, Shanghai, China). The cryostat liver tissue sections (10 μm) from rats were stained with 5 μM DHE at room temperature for 25 min. in a humidified chamber. The fluorescence images were obtained using a Zeiss 573–637 nm filter under a fluorescence microscope (Nikon Eclipse E800, Tokyo, Japan), and quantitative analysis was performed with ImageJ software (version 1.60; National Institutes of Health, Bethesda, MD, USA).

### Histological analysis and immunofluorescence staining

Livers were fixed in 10% neutral buffer formalin and embedded in paraffin. Tissue sections were cut at 3 μm and stained for haematoxylin–eosin (HE) using conventional methods. For hypothalamic immunostaining, rats were transcardially perfused with saline containing heparin (50 i.u./l), followed by 4% paraformaldehyde. Subsequently, all brains were removed, postfixed in 4% paraformaldehyde for 1 hr and then placed in phosphate‐buffered saline containing 30% sucrose. Brain sections were cut at 6 μm thickness using a cryostat at −20°C. After blocked with appropriate serum, sections were penetrated with 0.2% TritonX‐100 and incubated with primary rabbit anti‐POMC antibodies (1:200, sc‐365831, Santa Cruz Biotechnology, Inc., Dallas, TX, USA) and subsequently detected with FITC‐labelled goat anti‐rabbit secondary antibody (Invitrogen, Carlsbad, CA, USA). Images were captured using the Olympus FW1000 confocal microscope.

### Western blot analysis

Preparation of protein samples and Western blot analyses was performed as previously described 19. The liver homogenates were added with a lysis buffer and heated at 95°C for 2 min. Sample aliquots were subjected to sodium dodecyl sulphate polyacrylamide gel electrophoresis (SDS‐PAGE) and transferred onto polyvinylidene difluoride (PVDF) membranes (Millipore‐Linco, St Charles, MO, USA) for Western blot. Primary antibodies included anti‐β‐actin (1:3000, Bioworld Technology, Nanjing, China), anti‐NF‐κ B (p65) (1:2500, sc‐8008), anti‐phosphorylated NF‐κ B (p‐p65) (Ser536) (1:1000, sc‐136548), anti‐Iκ Bα (1: 1000, sc‐1643), anti‐AKT (1:2000, cst‐9272, Cell Signaling Technology, Inc., Beverly, MA, USA), anti‐phosphorylated AKT (Ser473) (1:1000, cst‐9271), anti‐STAT3 (1:2500, cst‐9139) and anti‐phosphorylated‐STAT3 (Tyr 705) (1:1000, cst‐9145). The bound antibodies were detected with horseradish peroxidase (HRP)‐conjugated secondary antibody. Bands were scanned, and a densitometer analysis was performed using Image J (National Institute of Health).

### Real‐time PCR

Total RNA was extracted from the hypothalamus and liver using Trizol reagent and immediately reverse transcribed into cDNA using High‐capacity cDNA Reverse Transcription kit according to the manufacturer's protocol (Invitrogen). Real‐time PCR was performed using an ABI 7900 HT Real‐Time PCR System (Applied Biosystems Inc., Foster City, CA, USA). Relative expression levels of each target gene to the internal control (GAPDH) were determined by 2^−∆∆CT^ method. The primer sequences used in the study are listed in Table S1.

### Statistical analysis

Data are expressed as mean ± S.E.M. *P* values were calculated using Student's *t*‐test and one‐way anova with Sidak multiple comparisons test as appropriate. *P* < 0.05 was considered statistically significant. SPSS22.0 (SPSS Inc., Chicago, IL, USA) was used for the statistical analyses.

## Results

### Peripheral insulin alleviated systemic metabolic disorders induced by LPS

**Figure 1:**
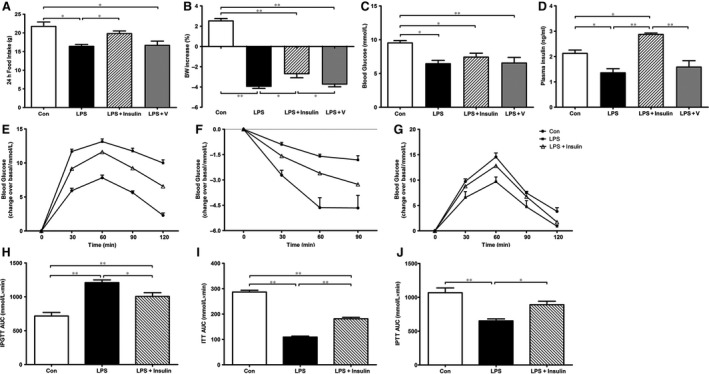
Insulin treatment can alleviate sepsis‐induced systemic metabolic disorders. Twenty‐four hours after treatment, metabolic parameters of rats were measurement. Twenty‐four‐hours food intake (**A**), BW increase (**B**), random blood glucose (**C**) and plasma insulin levels (**D**). All rats were subjected to IPGTT (**E**), ITT (**F**) and IPTT(**G**). Area under the curve (AUC) of each test was calculated and analysed (**H**–**J**). The data are presented as means ± S.E.M. *n* = 6 rats per group. *P < 0.05, **P < 0.01.

As shown in Figure 1, food intake, body weight (BW), blood glucose and plasma insulin levels were significantly decreased at 24 hrs after LPS injection when compared to Con (Fig. 1A–D). Importantly, in the LPS+Insulin group, rats showed a less reduction in body weight. In addition, results of ITT and IPTT in LPS+Insulin rats displayed significantly improved insulin sensitivity and enhanced hepatic glucose production (HGP) (Fig. 1E–J). Collectively, these results demonstrated LPS induced body weight losses, blood glucose decrease, insulin resistance and reduction in HGP. Peripheral insulin administration could attenuate these disorders of metabolism.

### The effect of insulin on LPS‐induced liver injury and activation of hepatic NF‐κB pathway

**Figure 2:**
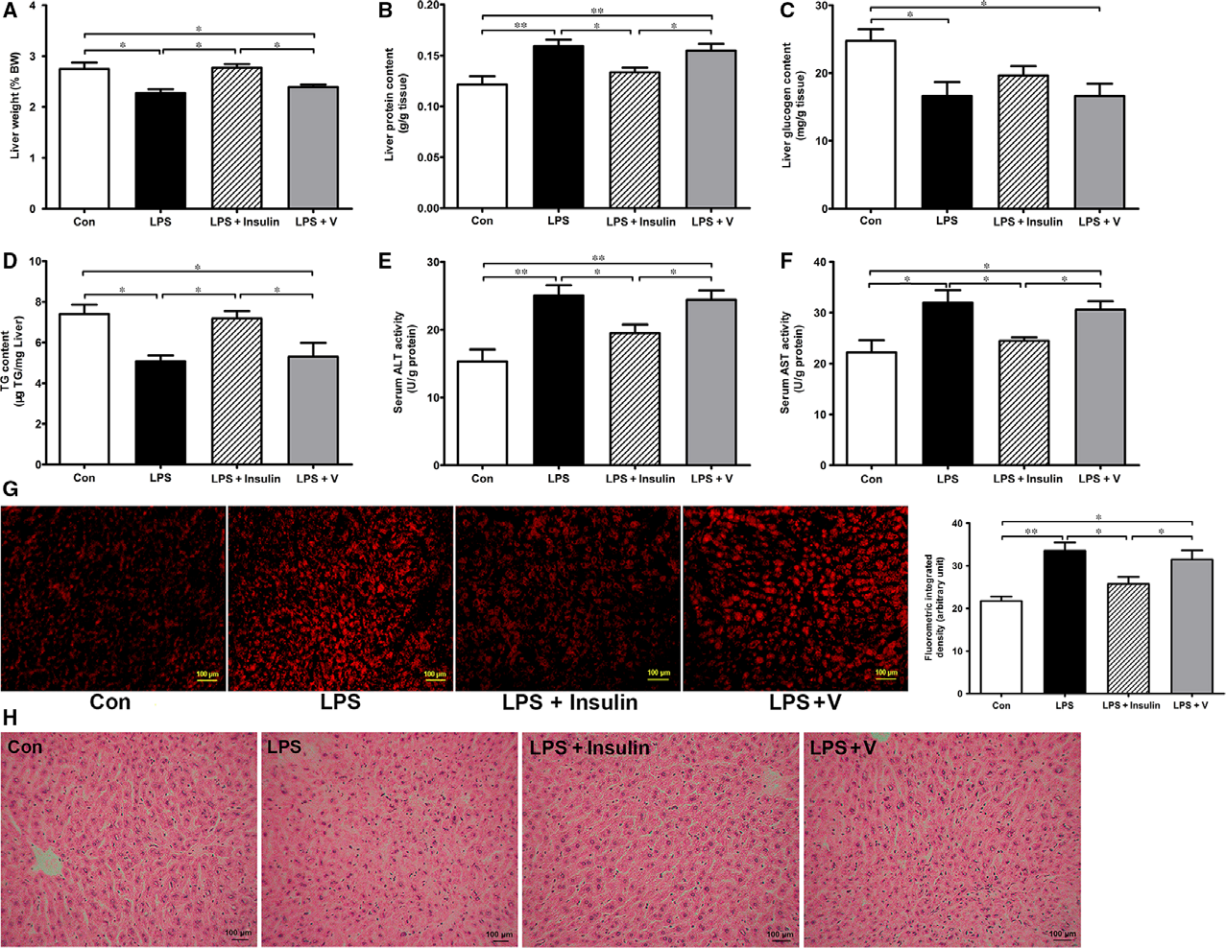
Insulin attenuates hepatic injury in LPS injected rats. At the end of study, all rats were euthanized for collection of liver tissue. Liver weight (**A**), protein content (**B**), glycogen content (**C**) and triglyceride (**D**) in rats. Serum alanine aminotransferase (ALT) (**E**) and aspartate aminotransferase (AST) (**F**) were measured by commercial kits. DHE staining in liver sections and fluorescence intensity analysis of different groups of rats (**G**). Original magnification ×100; Scale bar, 200 μm. Representative images of HE staining are shown (**H**). The data are shown as means ± S.E.M. *n* = 6 rats per group. *P < 0.05, **P < 0.01.

Earlier studies have demonstrated LPS‐induced acute liver injury 20, 21. Here, we found that LPS caused a significant decrease in liver weight, hepatic glycogen and TG content but an increase in liver protein content (Fig. 2A–D). Meanwhile, serum alanine aminotransferase (ALT) and aspartate aminotransferase (AST) were obviously elevated at 24 hrs after LPS injection (Fig. 2E and F), suggestive of impaired liver function. To investigate whether hepatic ROS was overproduced, liver sections were stained with DHE. As indicated in Figure 2G, the intensity of fluorescent was significantly enhanced after LPS injection and mitigated with insulin treatment. Furthermore, focal and massive necrosis in liver was observed in the LPS group and largely attenuated by insulin (Fig. 2H).

**Figure 3:**
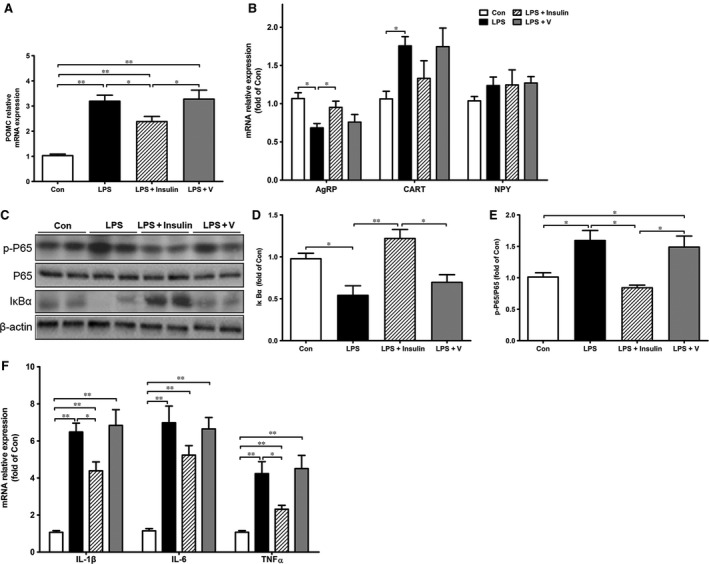
Effects of peripheral insulin on hypothalamic neuropeptides expression and hepatic inflammation signalling pathway. The mRNA expression of hypothalamic neuropeptide POMC (**A**), AgRP, CART and NPY (**B**). Hepatic inflammation signaling pathway proteins (p‐p65, p65 and IκBα) were determined by Western blotting (**C**) and were normalized by β‐actin or the total protein levels of p65 (**D**,** E**). Hepatic pro‐inflammatory cytokine (IL‐1β, IL‐6 and TNF‐α) expression was measured by real‐time PCR (**F**). ImageJ was used for quantifying the relative levels of proteins. β‐actin was used as internal control. Values are expressed as means ± S.E.M. *n* = 6 rats per group. *P < 0.05, **P < 0.01.

As indicated in Figure 3A and B, LPS induced a significant increase in POMC and peptide cocaine‐ and amphetamine‐regulated transcript (CART) expression and decrease in agouti‐related protein (AgRP) expression. However, these changes were attenuated by insulin. Next, we examined the activation of hepatic NF‐κB pathway. In the LPS group, phosphorylation of subunit p65 strongly increased and the content of IκBα was accordingly decreased when compared to the control (Fig. 3C–E). As expected, peripheral insulin treatment inhibited phosphorylation of p65 and increased protein level of IκBα (Fig. 3C–E). Besides, LPS induced higher mRNA expression of pro‐inflammatory cytokines in liver such as IL‐1β, IL‐6 and TNFα, which was markedly suppressed by insulin (Fig. 3F). Meanwhile, as shown in Table S2, IL‐6 was increased in LPS‐injected rats and decreased by insulin treatment. Plasma corticosterone and hypothalamic α‐MSH showed a similar change pattern.

### Insulin restored hepatic GNG in septic rats through STAT3 inactivation

**Figure 4:**
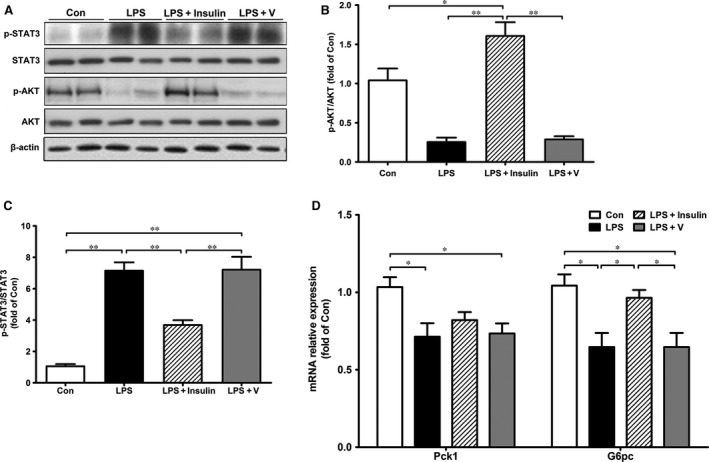
Effects of insulin on hepatic signalling pathway and gluconeogenesis in septic rats. Hepatic signalling pathway proteins (p‐STAT3, STAT3, p‐AKT and AKT) were determined by Western blotting (**A**), and the relative levels of protein were quantified by densitometric scanning using ImageJ (**B**,** C**). Expression of the key enzyme in hepatic gluconeogenesis (Pck1 and G6pc) was measured by real‐time PCR (**D**). β‐actin was used as internal control. All data are represented as means ± S.E.M. *n* = 6 rats per group. *P < 0.05, **P < 0.01.

As previous work has demonstrated that STAT3 plays a presumptive role in inhibiting GNG 22, we next investigated whether lower blood glucose was associated with higher hepatic STAT3 activity in sepsis rats. As shown in Figure 4A–C, phosphorylation of STAT3 (active form) significantly increased after LPS injection; however, this activation was diminished by insulin. Phosphorylation of AKT, a critical step in the insulin signalling pathway, was also determined, and no obvious changes were observed in livers. mRNA expression of Pck1 and G6pc further confirmed that hepatic GNG was inhibited in sepsis rats, which was partly restored by insulin treatment though the change of Pck1 was not significant (Fig. 4D).

### The effect of POMC knockdown on systemic metabolism of LPS‐treated rats

**Figure 5:**
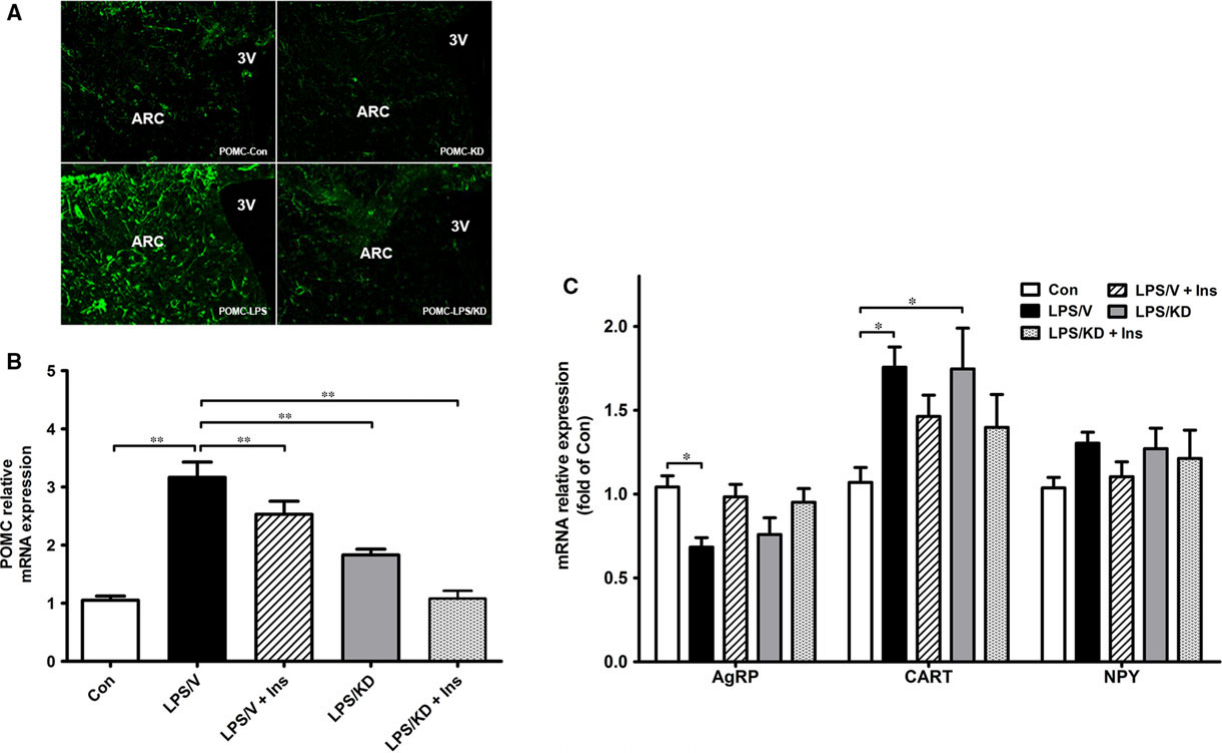
Hypothalamic neuropeptides expression in LPS‐treated rats after ARC injection of lentiviruses against POMC. Brain tissue was harvested for immunofluorescence staining as described in method. Representative images of POMC are shown (green) (**A**). mRNA expression of POMC and other hypothalamic neuropeptides in different groups of rats **(B, C)**. Reported values are relative to β‐actin. Data are expressed as means ± S.E.M. *P < 0.05, **P < 0.01.

As hypothalamus plays essential roles in regulating feeding behaviour and peripheral metabolism 23, we next investigated whether the effect of insulin on hepatic damage and systemic metabolism was mediated by hypothalamic POMC. We employed a lentiviral‐mediated delivery of shRNA to knock down POMC expression. The efficiency was confirmed by immunostaining and mRNA determination (Fig. 5A and B). LPS resulted in decreased AgRP and increased CART expression, but no difference was observed between LPS/V and LPS/KD group (Fig. 5C). These results suggested knockdown of POMC almost has no effect on the expression of other neuropeptides.

**Figure 6:**
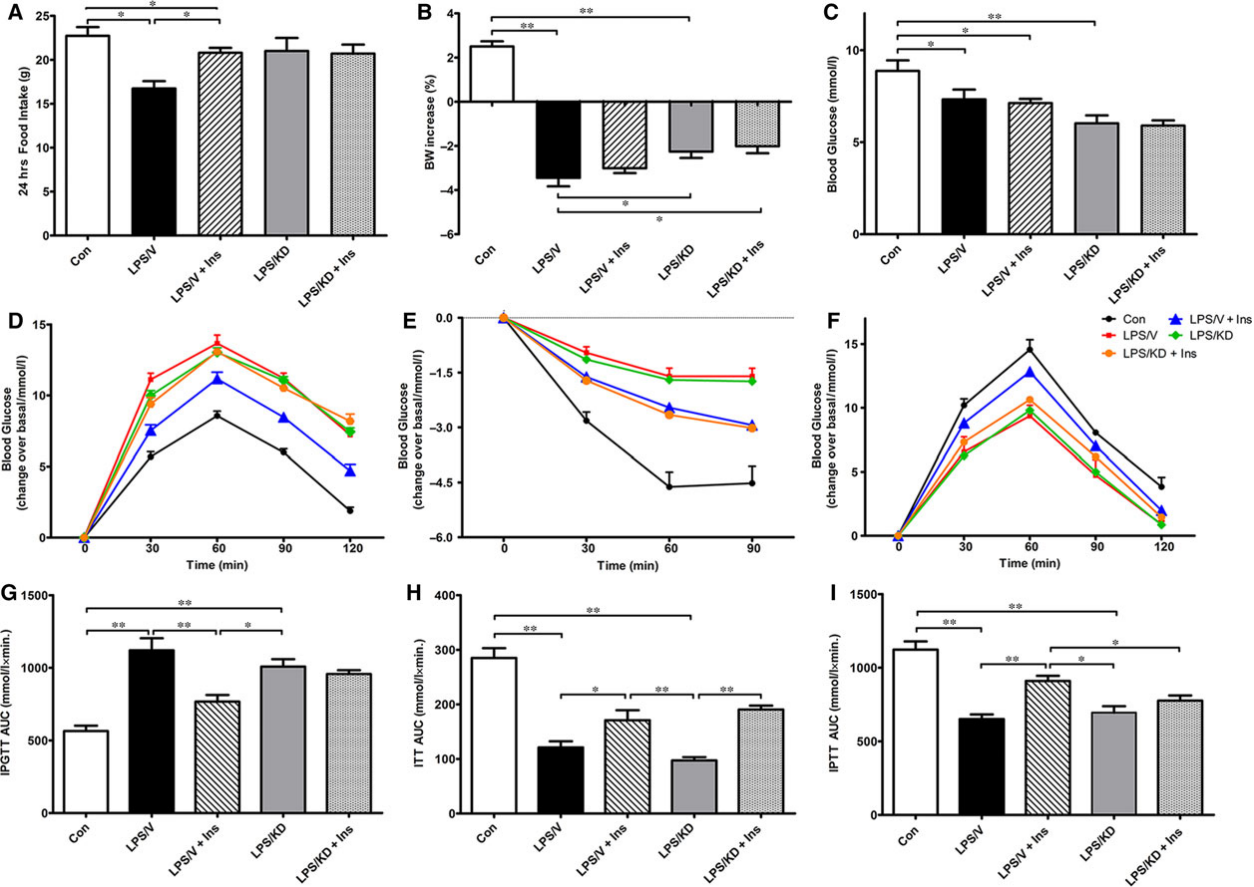
Effects of hypothalamic POMC knockdown on the systemic metabolism in septic rats. Twenty‐four hours after treatment, metabolic parameters of rats were measurement. Twenty‐four‐hours food intake (**A**), BW increase (**B**) and random blood glucose (**C**). All rats were subjected to IPGTT (**D**), ITT (**E**) and IPTT(**F**). Area under the curve (AUC) of each test was calculated and analysed (**G**‐**I**). Data are expressed as means ± S.E.M. *n* = 6 rats per group. *P < 0.05, **P < 0.01.

As shown in Figure 6A–C, LPS/KD rats showed higher food intake, less body weight loss as well as lower blood glucose when compared to LPS/V rats. However, no difference was observed between the LPS/KD and LPS/KD+Insulin group of rats. In IPGTT, ITT and IPTT analysis, insulin treatment largely attenuated insulin resistance and promoted hepatic GNG in sepsis rats. However, the latter was diminished by POMC knockdown (Fig. 6D–I), indicative of a critical role of hypothalamic POMC in insulin action on hepatic GNG regulation.

### The liver protective and NF‐κB pathway inhibiting activity of insulin was unaffected by hypothalamic POMC knockdown

**Figure 7:**
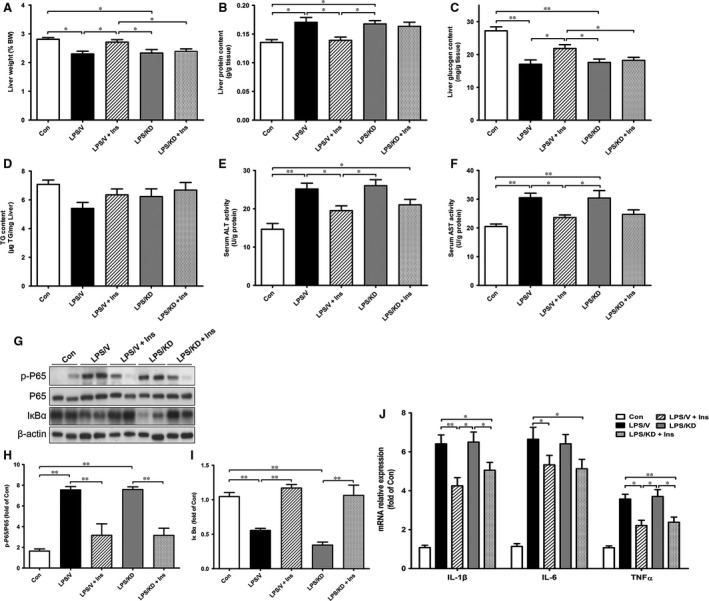
Knocking down hypothalamic POMC expression does not affect the protective effects of insulin on sepsis rats. At the end of study, all rats were euthanized for collection of liver tissue. Liver weight (**A**), protein content (**B**), glycogen content (**C**) and triglyceride (**D**) in different groups of rats. Serum alanine aminotransferase (ALT) (**E**) and aspartate aminotransferase (AST) (**F**) were measured by commercial kits. Hepatic NF‐κB pathway proteins were determined by Western blotting (**G**) and were normalized by β‐actin or the total protein levels (**H**,** I**). The expression of hepatic pro‐inflammatory cytokine was measured by real‐time PCR (**J**). ImageJ was used for quantifying the relative levels of proteins. β‐actin was used as internal control. Values are shown as means ± S.E.M. *P < 0.05, **P < 0.01.

Hepatic injury and NF‐κB pathway activity in POMC knockdown rats were measured. As shown in Figure 7A–D, liver weight and glycogen content was increased in LPS/V+Ins group when compared to LPS/V; however, both changes mentioned above were blocked by POMC knockdown. Liver protein or TG content did not show a significant difference when POMC was knocked down in LPS/V+Ins rats. Additionally, serum ALT, AST content as well as hepatic NF‐κB pathway had no significant difference between LPS/V+Ins and LPS/KD+Ins group (Fig. 7E–I). Similarly, expression of IL‐1β, IL‐6 and TNFα in POMC knockdown rats was unaffected (Fig. 7J). Therefore, the liver protective and anti‐inflammatory effect of insulin in sepsis rats could be independent of hypothalamic POMC. Although expression of POMC was blocked, LPS still increased plasma IL‐6, corticosterone as well as hypothalamic α‐MSH, which were reduced with insulin treatment (Table S3).

### Knockdown of hypothalamic POMC reversed the enhancement of hepatic GNG in insulin‐treated rats

**Figure 8:**
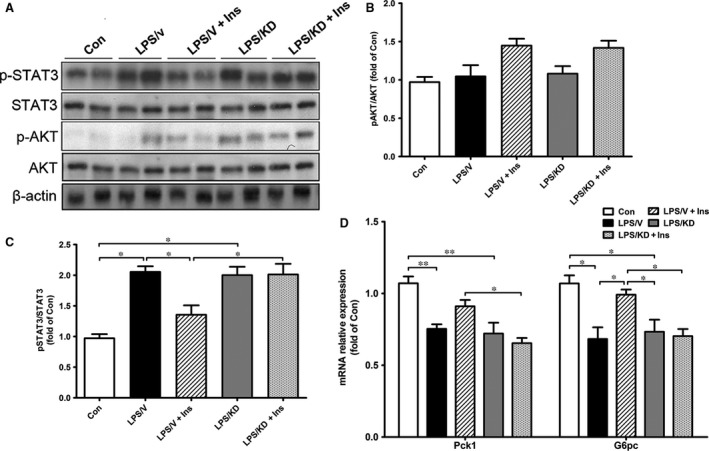
Knockdown of hypothalamic POMC diminished insulin‐induced hepatic STAT3 inhibition and enhanced gluconeogenesis in LPS‐treated rats. Hepatic signalling pathway proteins (p‐STAT3, STAT3, p‐AKT and AKT) were determined by Western blotting (**A**), and the relative levels of protein were quantified by densitometric scanning by ImageJ (**B**,** C**). Expression of the essential enzymes in hepatic gluconeogenesis (Pck1 and G6pc) was measured by real‐time PCR (**D**). Values are relative to β‐actin. All data are represented as means ± S.E.M. *n* = 6 rats per group. *P < 0.05, **P < 0.01.

With regard to the impact of POMC knockdown on hepatic GNG in sepsis rats, we next measured expression of STAT3 and key enzymes involved in GNG *via* Western blot or quantitive PCR analysis. As shown in Figure 8A–C, STAT3 protein was largely phosphorylated in liver of LPS/V rats, which was significantly mitigated by insulin treatment. However, in septic rats lack of POMC expression in hypothalamus, insulin failed to suppress the phosphorylation of STAT3. Besides, insulin‐induced phosphorylation of AKT was not affected by POMC knockdown. As expected, insulin increased expression of Pck1 and G6pc when compared to those in the LPS/V group; however, both of which were diminished in LPS/KD+Ins rats although the change of Pck1 did not achieve significance (Fig. 8D). Collectively, these results suggested a POMC‐depended effect of insulin on restoring hepatic GNG in sepsis rats.

**Figure 9:**
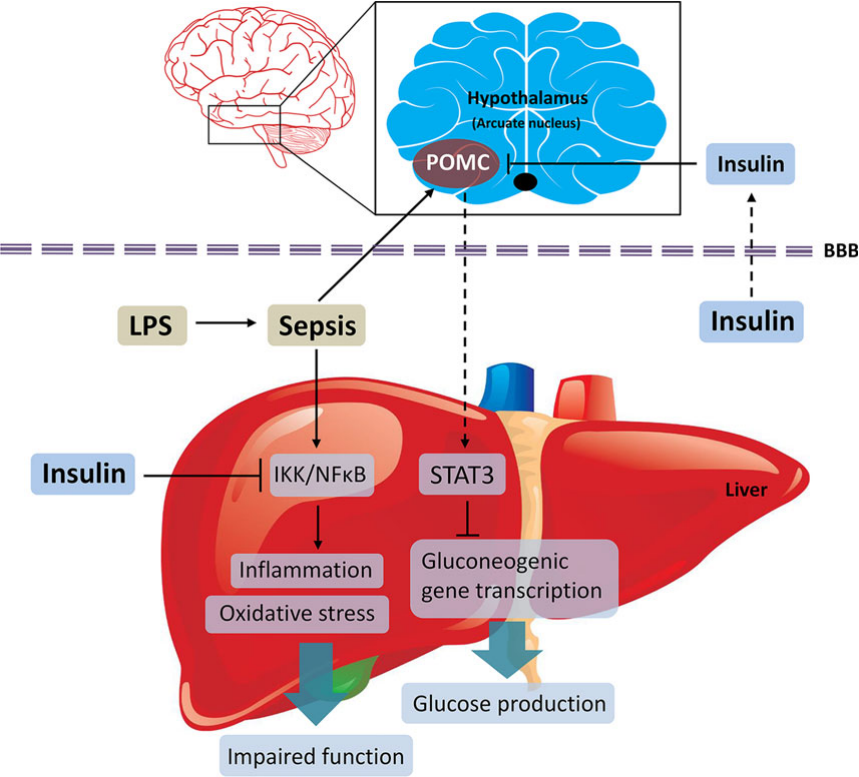
A schematic illustration of mechanisms for the effect of insulin on hepatic function impairment and GNG in septic rats. Hepatic IKK/NF‐κB pathway mediates the anti‐inflammatory effect of insulin in sepsis rats, and peripheral insulin treatment may improve hepatic GNG by inhibiting STAT3 phosphorylation through reducing hypothalamic POMC expression.

## Discussion

In this study, we demonstrated that peripheral insulin administration could inhibit the activation of hepatic NF‐κB pathway and protect against the liver injury in sepsis rats. More importantly, the repression of hepatic GNG induced by LPS was significantly restored by insulin treatment probably through inactivation of hepatic STAT3. Furthermore, the knocking down experiment by a central lentiviral approach indicated that hypothalamic POMC expression contributed to the enhancement of hepatic GNG in sepsis rats, but was not required for the anti‐inflammatory action of insulin.

IKKβ/NF‐κB is a crucial pathway in regulating the expression of genes associated with inflammation and immune response 24. Numerous studies have suggested activation of NF‐κB in peripheral or/and central tissues is essential for infectious and metabolic diseases, such as sepsis, anorexia, obesity and diabetes 25, 26, 27, 28. A previous report has demonstrated that hepatic Kupffer cells are responsible for producing several early pro‐inflammatory mediators in sepsis, such as IL‐1β, TNF‐α, IL‐6 and IFN‐γ 29. Surprisingly, IL‐6 could also promote hepatic cell survival and breakdown of glycogen 30, 31, which was in line with the LPS‐induced decrease in liver glycogen content in our study. The insulin treatment significantly decreased the level of ALT, AST and ROS production in liver, suggestive of alleviating hepatic injury/dysfunction. Coincidentally, insulin strongly inhibited NF‐kB activity and expression of pro‐inflammatory cytokines such as IL‐1β, TNF‐α and IL‐6 in liver as shown in our results, which maybe further facilitate the attenuation of liver damage. In addition, hepatic GSK‐3β inactivation also contributed to the protective effects of insulin 10. Because α‐MSH could prevent hepatic inflammation through inhibiting the production of related cytokines 32, the increased POMC expression in the hypothalamus might be a compensatory response under the condition of sepsis.

Besides inflammatory response and injury in multiple organs, administration of LPS usually resulted in obvious metabolic disorders, including hyperglycaemia, hypoglycaemia, insulin resistance and other manifestations 4, 33, 34. In fact, noninsulin‐mediated glucose uptake by tissues, such as spleen, ileum, liver and lung, during severe infection could result in enhanced glucose utilization even hypoglycaemia 34. Under this circumstance, hepatic glycogenolysis and GNG should be the major source of glucose to maintain systemic metabolism at homoeostasis. However, previous studies have shown diminished activity and/or expression of PEPCK/G6pase, the rate‐limiting enzyme in hepatic GNG 4, 34, which were consistent with our present findings. As indicated in results, LPS injection caused an apparent reduction in blood glucose, as well as insulin and pyruvate tolerance impairment, accompanied with inhibited mRNA expression of Pck1 and G6pc in liver. As expected, treatment with insulin significantly mitigated injurious changes above in sepsis rats. Nevertheless, mechanisms underlying these protective effects of insulin are still confusing till now. Dugo *etc*. observed an alleviation of liver dysfunction with insulin treatment despite the persistent hypoglycaemia during sepsis, suggesting that insulin may elicit its beneficial effects independently of blood glucose 10. Indeed, no significant changes in glucose blood were observed while sepsis rats received insulin treatment.

Recently, researchers have demonstrated that peripheral LPS injection can cause a local inflammation in the hypothalamus, and inhibiting hypothalamic NF‐κB pathway may affect food intake and body weight by regulating the expression of key neuropeptides, especially POMC and AgRP 27. Consistently, we found that insulin treatment significantly reduced body weight losses and POMC expression. On the contrary, expression of AgRP, the orexigenic neuropeptide, was up‐regulated by insulin administration. Based on these results, we questioned whether hypothalamic neuropeptides contributed to the beneficial effects on systemic metabolism even though insulin was peripherally administrated. Therefore, we blocked the elevation of POMC expression after LPS injection using a lentiviral vector of shRNA. Obviously, in the knockdown group of rats, body weight loss was further attenuated with or without insulin treatment when compared to the Con and LPS/V group. However, POMC knockdown significantly reversed effect of insulin on hepatic STAT3 and the downstream Pck1 and G6pc expression, as well as liver glycogen content. Besides, insulin‐induced improvement of pyruvate tolerance was also diminished in the LPS/KD+Ins group. More interestingly, we found that POMC knockdown had no significant impact on the activities of hepatic enzyme markers (ALT and AST) and NF‐κB pathway with or without insulin treatment in sepsis rats. These results indicated hypothalamic POMC expression was essential for improving hepatic GNG with peripheral insulin treatment, and the anti‐inflammatory action of insulin was probably independent of POMC in sepsis rat models.

Plenty of studies have recognized the hypothalamus as an essential regulator of diverse body functions including hepatic GNG 23, and the ARC is privileged among these nuclei 35, 36. POMC neurons of ARC are emerging critical roles in the modulation of hepatic glucose production (HGP) 17, 37, 38. However, the exact mechanism of POMC mediating hepatic GNG still remain speculative, although hepatic CREB activity is considered a key mediator 39. Recent evidence indicates that melanocortin system, may regulate HGP through connections to autonomic centres and MC4R signalling in cholinergic preganglionic sympathetic neurons probably mediates HGP restraint 23, 40. In consideration of these results, it seems reasonable to speculate that changed POMC expression is also implicated in the impairment of hepatic GNG in the context of sepsis. Indeed, our data demonstrated a causative involvement of POMC in the maintenance of glucose homoeostasis as decreased POMC expression and plasma α‐MSH levels by treatment of insulin restored hepatic GNG in septic rats, which is coincident with the finding that reduced melanocortin tone in α‐MSH defective mice is associated with increased hepatic GNG 39. Furthermore, several studies have reported that central insulin could activate POMC neurons and promote HGP *via* insulin receptors 41, 42; however, Obici *et al*. showed that central injection of insulin diminished hepatic glucose output 43. Thus, the relative importance of hypothalamic insulin signalling for the overall modulation of hepatic glucose homoeostasis requires more assessments in future work. In the present study, only POMC knockdown had no significant effect on the expression of hepatic STAT3 and key enzymes involved in GNG. However, the beneficial effect of insulin on hepatic GNG was diminished when POMC was knocked down. It is worth noting that although insulin was not centrally administrated in our experiments, peripherally circulating insulin crosses the blood–brain barrier (BBB) in proportion to serum insulin levels *via* a saturable mechanism. POMC neurons can also sense the fluctuation of insulin in the blood, owing to its close contact with the median eminence, where BBB is incomplete 44, and the involvement of highly expressed insulin receptor‐mediated transport mechanisms. Besides the limitation of peripheral administration of insulin, collectively, our new findings indicate that hypothalamic POMC directly mediated the peripheral insulin action on hepatic GNG, although potential effects of insulin on other hypothalamic areas cannot be excluded.

In summary, our work shows that insulin administration displays critical liver and glucose metabolism protective roles in sepsis rat models, possibly through suppression of NF‐κB pathway and neuropeptide POMC‐mediated inhibition of STAT3 in liver. However, further studies are essential to determine more molecular mechanisms how insulin interacts with POMC neurons to regulate HGP in sepsis.

## Conflict of interest

The authors confirm that there are no conflicts of interest.

## Supporting information


**Table S1.** The primer sequences for RT‐PCR analysis.

**Table S2.** Plasma IL‐6, corticosterone and hypothalamic α‐MSH content in different groups of rats.

**Table S3**. Effects of hypothalamic POMC knockdown on plasma IL‐6, corticosterone and hypothalamic α‐MSH levels of experimental rats.
