# Supplementary material for: Hypothalamic POMC expression is required for peripheral insulin action on hepatic gluconeogenesis through regulating STAT3 in sepsis rats
Source: J Cell Mol Med. 2017 Dec 28;22(3):1696–707. doi: 10.1111/jcmm.13449 (PMC5824389; doi:10.1111/jcmm.13449)
Supplement: Supplementary file 1 — Table S1. The primer sequences for RT‐PCR analysis. [file JCMM-22-1696-s001.docx]

| Gene name | Primer sequence (5’-3’) | Accession No. |
| --- | --- | --- |
| GAPDH | F: GCAAGTTCAACGGCACAG  R: GCCAGTAGACTCCACGACAT | [NM_017008.4](https://www.ncbi.nlm.nih.gov/nuccore/NM_017008.4) |
| Pck1 | F: GAGATCATCTCCTTCGGAAGCG  R: TTAGTTATGCCCAGGATCAGCATG | [NM_198780.3](https://www.ncbi.nlm.nih.gov/nuccore/NM_198780.3) |
| G6pc | F: AACGTCTGTCTGTCCCGGATC-TA  R: CCTCTGGAGGCTGGCATTGTA | [NM_013098.2](https://www.ncbi.nlm.nih.gov/nuccore/NM_013098.2) |
| POMC | F: CCTCCTGCTTCAGACCTCCA  R: GGCTGTTCATCTCCGTTGC | [NM_139326.2](https://www.ncbi.nlm.nih.gov/nuccore/NM_139326.2) |
| AgRP | F: TGAAGGGCATCAGAAGGT  R: CACAGGTCGCAGCAAGGT | [NM_033650.1](https://www.ncbi.nlm.nih.gov/nuccore/NM_033650.1) |
| CART | F: CCGAGCCCTGGACATCTA  R: GGAATGCGTTTACTCTTGAGC | [NM_017110.1](https://www.ncbi.nlm.nih.gov/nuccore/NM_017110.1) |
| NPY | F: GTGTTTGGGCATTCTGGCTG  R: AGTGTCTCAGGGCTGGATCT | [NM_012614.2](https://www.ncbi.nlm.nih.gov/nuccore/NM_012614.2) |
| IL-1β | F: TTCAAATCTCACAGCAGCAT  R: AGGTCGTCATCATCCCAC | [NM_031512.2](https://www.ncbi.nlm.nih.gov/nuccore/NM_031512.2) |
| IL-6 | F: AAGGACCAAGACCATCCAAC  R: ACCACAGTGAGGAATGTCCA | [NM_012589.2](https://www.ncbi.nlm.nih.gov/nuccore/NM_012589.2) |
| TNF-α | F: CCACGCTCTTCTGTCTACTG  R: GCTACGGGCTTGTCACTC | [NM_012675.3](https://www.ncbi.nlm.nih.gov/nuccore/NM_012675.3) |

**Supplementary Table 1. The primer sequences for RT-PCR analysis.** Accession numbers are from the GenBank database.
