# Supplementary material for: Hypothalamic POMC expression is required for peripheral insulin action on hepatic gluconeogenesis through regulating STAT3 in sepsis rats
Source: J Cell Mol Med. 2017 Dec 28;22(3):1696–707. doi: 10.1111/jcmm.13449 (PMC5824389; doi:10.1111/jcmm.13449)
Supplement: Supplementary file 2 — Table S2. Plasma IL‐6, corticosterone and hypothalamic α‐MSH content in different groups of rats. [file JCMM-22-1696-s002.docx]

| **Group** | **Con** | **LPS** | **LPS+Insulin** | **LPS+V** |
| --- | --- | --- | --- | --- |
| IL-6 (pg/ml) | 20 ± 8 | 214 ± 19^a,b^ | 155 ± 13^a^ | 226 ± 31^a,b^ |
| Corticosterone (ng/ml) | 43 ± 9 | 167 ± 25^a^ | 102 ± 15^a^ | [142](https://www.ncbi.nlm.nih.gov/nuccore/NM_198780.3) ± 21^a^ |
| α-MSH (ng/mg protein) | 0.712 ± 0.013 | 0.895 ± 0.017^a,b^ | 0.812 ± 0.013^a^ | [0.93](https://www.ncbi.nlm.nih.gov/nuccore/NM_013098.2)8 ± 0.021^a,b^ |

**Supplementary Table 2. Plasma IL-6, corticosterone and hypothalamic** **α-MSH content in different groups of rats.** IL-6 and α-MSH were measured by ELISA according to the manufacturer’s instructions. Corticosterone was measured by RIA. Statistical analyses were performed by one-way analysis of variance (ANOVA) followed by Sidak multiple comparisons test.Values are represented as mean ± SEM. ^a^*P* < 0.05 versus Con, ^b^*p* < 0.05 versus LPS+Insulin.
