# Supplementary material for: Hypothalamic POMC expression is required for peripheral insulin action on hepatic gluconeogenesis through regulating STAT3 in sepsis rats
Source: J Cell Mol Med. 2017 Dec 28;22(3):1696–707. doi: 10.1111/jcmm.13449 (PMC5824389; doi:10.1111/jcmm.13449)
Supplement: Supplementary file 3 — Table S3. Effects of hypothalamic POMC knockdown on plasma IL‐6, corticosterone and hypothalamic α‐MSH levels of experimental rats. [file JCMM-22-1696-s003.docx]

| **Group** | **Con** | **LPS/V** | **LPS/V+Ins** | **LPS/KD** | **LPS/KD+Ins** |
| --- | --- | --- | --- | --- | --- |
| IL-6 (pg/ml) | 25 ± 9 | 227 ± 24^a^ | 161 ± 16^a,b^ | 251 ± 32^a^ | 124 ± 15^a,b^ |
| Corticosterone (ng/ml) | 51 ± 9 | 179 ± 35^a^ | 122 ± 18 | 152 ± 23^a^ | 114 ± 13 |
| α-MSH (ng/mg protein) | 0.624 ± 0.009 | 0.865 ± 0.014^a^ | 0.792 ± 0.014^a,b,c^ | 0.883 ± 0.020^a^ | [0](https://www.ncbi.nlm.nih.gov/nuccore/NM_013098.2).801 ± 0.016^a,b,c^ |

**Supplementary Table 3. Effects of hypothalamic POMC knockdown on plasma IL-6, corticosterone and hypothalamic α-MSH levels of experimental rats.** IL-6 and α-MSH were measured by ELISA according to the manufacturer’s instructions. Corticosterone was measured by RIA. Statistical analyses were performed by one-way ANOVA followed by Sidak multiple comparisons test.Values are represented as mean ± SEM. ^a^*P* < 0.05 versus Con, ^b^*p* < 0.05 versus LPS/V, ^c^*p* < 0.05 versus LPS/KD.
